# Morphology and Biochemistry of Ovulation

**DOI:** 10.1055/s-0041-1731379

**Published:** 2021-07-27

**Authors:** Sebastião Freitas de Medeiros, Bruna Barcelo Barbosa, Matheus Antonio Souto de Medeiros, Márcia Marly Winck Yamamoto

**Affiliations:** 1 Department of Gynecology and Obstetrics, Faculdade de Medicina, Universidade Federal do Mato Grosso, Cuiabá, Mato Grosso, MT, Brazil; 2 Instituto Tropical de Medicina Reprodutiva, Cuiabá, Mato Grosso, MT, Brazil

**Keywords:** granulosa cells, follicle-stimulating hormone, luteinizing hormone, oocytes, ovarian follicle, células da granulosa, hormônio folículo-estimulante, hormônio luteinizante, oócitos, folículo ovariano

## Abstract

The process of ovulation involves multiple and iterrelated genetic, biochemical, and morphological events: cessation of the proliferation of granulosa cells, resumption of oocyte meiosis, expansion of cumulus cell-oocyte complexes, digestion of the follicle wall, and extrusion of the metaphase-II oocyte. The present narrative review examines these interrelated steps in detail. The combined or isolated roles of the follicle-stimulating hormone (FSH) and luteinizing hormone (LH) are highlighted. Genes indiced by the FSH genes are relevant in the cumulus expansion, and LH-induced genes are critical for the resumption of meiosis and digestion of the follicle wall. A non-human model for follicle-wall digestion and oocyte release was provided.

## Resumo

O processo de ovulação envolve modificações genéticas, bioquímicas e morfológicas múltiplas e interrelacionadas: suspensão da proliferação das células da granulosa, reinício da meiose do oócito, expansão das células do complexo cumulus-oócito, digestão da parede folicular, e extrusão do oócito. Esta revisão narrativa examina em detalhes cada um desses eventos e os principais genes e proteínas envolvidos. Mais importante, a ação combinada ou isolada do hormônio folículo-estimulante (HFE) e do hormônio luteinizante (HL) é destacada. Detalha-se o papel do HFE na expansão do cumulus e do HL na digestão da parede folicular, permitindo a extrusão do oócito na superfície ovariana. Proveu-se um modelo não humano para explicar a digestão da parede folicular.

## Introduction

Ovulation is the term used to define the ovarian release of the female mature gamete that is ready to be fertilized. The process of ovulation includes a series of morphological and biochemical events within the preovulatory follicle. Several genes are activated in the ovarian environment, leading to enzymatic and structural transformations under the influence of gonadotropins and sex steroids that are modulated by several growth factors. All of these events ensure that the oocyte becomes likely to be fertilized and extruded on the ovarian surface to form the corpus luteum. 1 The clinical marker of the beginning of the reproductive cycle responsible for the maturation and extrusion of the oocyte is menstruation. In regular cycles, at intervals of 24 to 38 days, 2 ovulation occurs mid-cycle, at around the 14th day. In this scenario, in an orchestrated way, the follicle-stimulating hormone (FSH), and the luteinizing hormone (LH) actively participate in the events that ensure ovulation, mostly through activation of multiple genes in theca and granulosa cells. The present review aims to examine the basic mechanisms of ovulation and describe the morphological and molecular events interconnected during the ovulatory process.

## Methods

We searched for articles published in English in the PubMed and Google Scholar databases. The keywords were as follows: *menstrual cycle* , *menstrual cycle physiology* , *folliculogenesis* , *theca cells* , *granulosa cells* , *oocyte* , *oocyte-cumulus complex* , *follicular wall digestion* , *cumulus-oocyte-complex expansion* , *oocyte maturation* , *gene expression* , *FSH* , *LH* , and *progesterone receptor* . We expanded the search to the references of the retrieved articles.

### Follicular Dynamics and Folliculogenesis

**Fig. 1:**
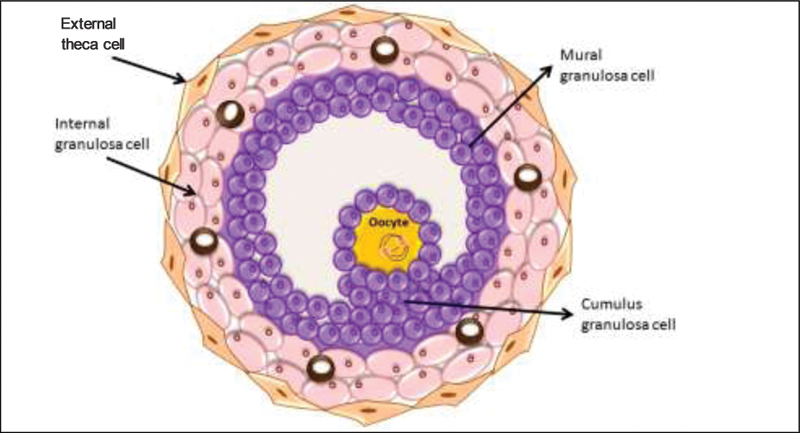
Structure of the periovulatory follicle showing internal and external theca cell layers, granulosa cells, and the oocyte.

**Fig. 2:**
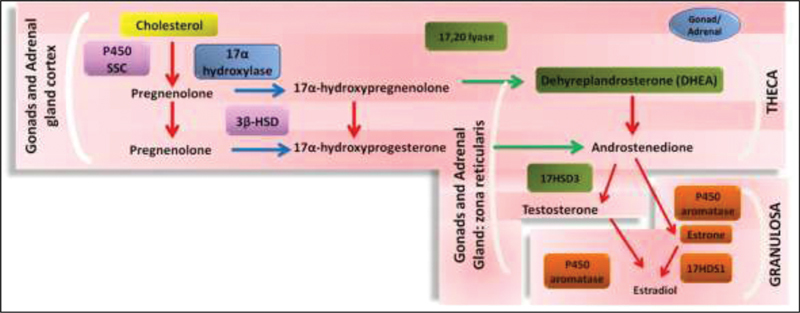
Scheme showing the steroidogenesis of theca and granulosa ovarian cells. Abbreviations: P450ssc, cytochrome P450 for cleavage of cholesterol side chains; 3-βSHD, 3 β hydroxysteroid dehydrogenase; 17-HSD3, 17-hydroxysteroid dehydrogenase. **Source:** Medeiros et al. 7

The more advanced stages of follicle development are characterized by the appearance of intercellular space filled by antral fluid. At this stage, the granulosa cells are differentiated into two distinct populations: cumulus cells, which are those closely linked to the oocyte, and wall or mural granulosa cells, which internally line the follicular wall. Although these two cell types share a common origin, there are differences in the production of transcribers and proteins. 3 At the end of follicular development, the FSH and estradiol promote the expression of the LH receptor (LHR) in granulosa cells. Most LH molecules bind to mural granulosa cells rather than to cumulus cells. 4 Cumulus cells provide energy input to the oocyte, controlling its growth and metabolism. 5 On the other hand, mural granulosa cells are responsible for steroid synthesis and differentiation in luteum cells after ovulation. 6 Cumulus granulosa cells play a smaller role regarding the function of the corpus luteum. Follicular architecture is provided by the inner and outer theca-cell layers ( Fig. 1 ). The theca cells, provided with LHR, are responsible for the capture of the substrate cholesterol and its enzymatic conversion into androgens, mainly testosterone (T) and androstenedione (A4). In turn, granulosa cells, which are adjacent to the theca cells, capture A4 and T and, by the action of the aromatase enzyme, convert them into estrone and estradiol respectively ( Fig. 2 ). 7

Folliculogenesis begins with the formation of the primordial follicle, and ends with the preovulatory follicle. 8 The FSH, released by the anterior pituitary gland, promotes the recruitment of follicular waves that, in response, secrete estradiol and inhibin. When synthesized, these hormones modulate the release of pituitary FSH and LH in a pulsatile way. At the end of folliculogenesis, the preovulatory peaks of FSH and LH induce a complex sequence (or even a concurrence) of events: oocyte maturation, cumulus cell expansion, follicular wall digestion, and release of the cumulus-oocyte complex. 9

### Ovulation Process

#### Genetic Aspects Determining Ovulation

The ovulation process occurs in a coordinated and interrelated way in five complex steps: interruption of granulosa cell proliferation, resumption of meiosis, expansion of the cumulus with oocyte release inside the antrum, lysis of the follicular wall, and oocyte extrusion at the metaphase II (MII) stage. In mammals, oocytes are stationed in meiosis I at prophase I. The resumption of meiosis I occurs during puberty as a result of the gonadotropic stimulus in follicles in the preovulatory stage, culminating in the rupture of the germ vesicle. 10 The increase in the concentrations of LH and FSH in the mid-cycle in the presence of the preovulatory follicle, now provided with LHR in granulosa cells, promotes the activation of several genes that encode the synthesis of various proteins. This process is similar to inflammatory processes. 11 The LH activates cyclase, resulting in intracellular increases in cyclic adenosine monophosphate (cAMP) that activate cAMP-dependent kinases and the expression of the hyaluronic synthase 2 (HAS-2) and cyclooxygenase 2 (COX-2) enzymes, the tumor necrosis factor-inducible gene 6 protein (TSG-6), pentraxin 3 (PTX-3), and genes of the epidermal growth factor (EGF)-like family, such as amphiregulin (AREG), epiregulin (EREG), and betacellulin (BTC). 12 13 14 Tissue rearrangement occurs as a result of the activation of these genes participating in the cascade of ovulation events.

#### The Role of the Follicle-stimulating Hormone

**Fig. 3:**
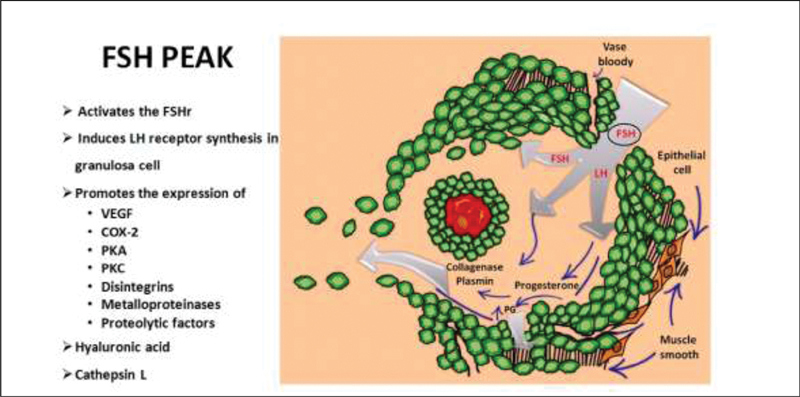
Biochemical events initiated by the follicle stimulating hormone (FSH) in the preovulatory follicle. Abbreviations: VEGF, vascular endothelial growth factor; COX-2, cyclaoxygenase-2; PKA, protein kinase A; PKC, protein kinase C.

Periovulatory gene expression induced by the FSH in cumulus cells plays a minor but necessary role in the mediation of ovulation ( Fig. 3 ). The occurrence of the FSH peak activates its own receptor (FSHR), stimulates the expression of steroidogenic factors, and induces LHR synthesis in granulosa cells. Such functions of the FSHR are related to the FSH activation of cAMP synthesis, and are triggered mainly through the expression of protein kinases A (PKA) and C (PKC) enzymes in granulosa cells. 15 The FSH activates the phosphatidylinositol 3-kinase/protein kinase B (PI3K/Akt) pathway to mediate cell survival and granulosa proliferation, including the expression of the vascular endothelial growth factor (VEGF) gene, and it activates extracellular-regulated kinase (ERK) signaling in mural granulosa and cumulus cells, facilitating cumulus expansion. 16 The FSH may also induce COX-2 and other prostaglandin synthases through cAMP/PKA activation. 17 Activation of the COX-2 gene results mainly in prostaglandin F-α (PGF2α) that induces changes in the gene expression of the cumulus-oocyte-complex, which is critical for cumulus-oocyte-complex expansion. 18 Additionally, the FSH induces the expression of genes belonging to the family of disintegrin and metalloproteinases (A disintegrin and metalloproteinase with thrombospondin motifs, ADAMTS), molecules relevant in the process of cleavage of the extracellular matrix ( Fig. 3 ). It seems that these proteins are the main regulators of the release of EGF-like proteolytic factors in a soluble form (AREG, EREG, and BTC), 19 which activate the EGF receptor tyrosine kinase and the extracellular signal-regulated kinase (ERK) involved in cumulus expansion. 13 Metalloproteinases ADAMTS-1, ADAMTS-4, ADAMTS-5, and ADAMS-16, genes expressed in granulosa cells, are involved in the dissociation of the cumulus-oocyte complex and in the formation of the corpus luteum. 20 21 Then, the FSH, in the same way as in the mucification of the cumulus, plays a role with the LH in the synthesis of enzymes responsible for the digestion of the follicle wall.

#### The Role of the Luteinizing Hormone

**Fig. 4:**
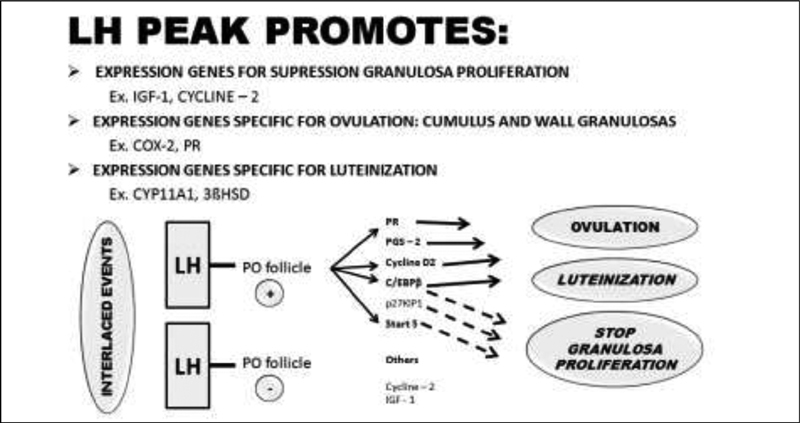
Expression of several genes induced by the luteinizing hormone (LH) peak. **Source:** Richards et al. 23 Abbreviations: PO, preovulatory; PR, progesterone receptor; PGS, prostaglandins; C/EBPβ, *CAAT* enhancer-binding protein β; p27KIP1, cyclin-dependent kinase inhibitor 1B; Start 5, steroidogenic enzymes; IGF, insulin growth factor; COX, cyclooxygenase.

The role the LH in the ovulation process is complex and fundamental for the resumption of meiosis, loosening of the cumulus cells, and rupture of the follicle. 22 With the peak of the LH, the messenger ribonucleic acid (mRNA) for the progesterone receptor (PR) as well as other genes is now transcribed into the granulosa cells of preovulatory follicles ( Fig. 4 ). 23 The PR has an indirect influence on the synthesis of proteolytic enzymes cathepsin L and ADAMTS-1, which together play a role in tissue degradation and the remodeling of the extracellular matrix at the apex of the preovulatory follicle until ovulation occurs. 24 The LH peak, modulated by AMP, participates in the process of suppression of the proliferation of granulosa cells, and restarts meiosis, dissociation of the granulosa, digestion of the follicle wall, and luteinization.

### Biochemical Aspects Determining Ovulation

#### Mucification and Cumulus Expansion

**Fig. 5:**
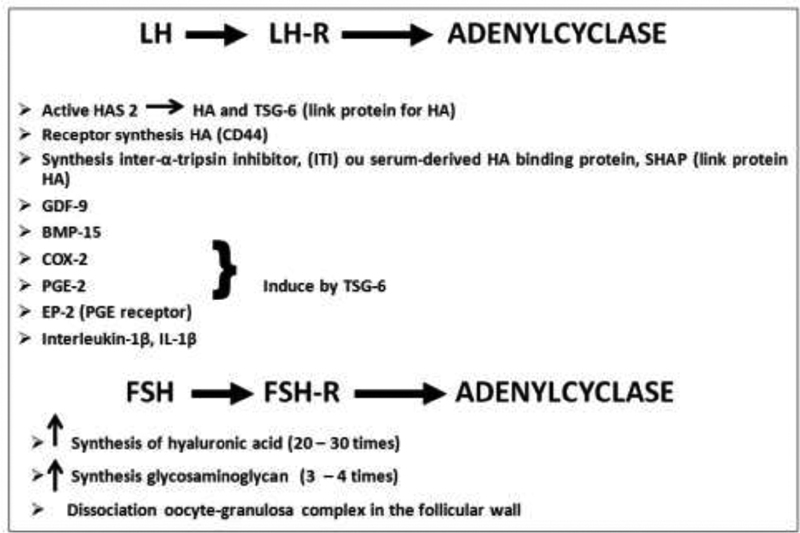
Combined actions of the FSH and LH in the expansion of oocyte-cumulus cells. **Source:** Richards et al. 23 Abbreviations: HA, hyaluronic acid; GDF, growth defferentation factor; BMP, bone morphogenetic protein; PE, prostaglandin E receptor.

The genetic and biochemical events responsible for cumulus mucification are summarized in Fig. 5 . 23 The matrix on which the cumulus cells move has three major components: hyaluronic acid (HA) and two HA binding proteins, TSG-6, and inter-α-trypsin inhibitor (ITI). 9 25 Induced by the peaks of FSH and LH, HAS-2 is the main enzyme responsible for the synthesis of arachidonic acids and HAs in the cumulus-oocyte complex, and, in synergy with COX-2, causes the synthesis of prostaglandins (PGs) from arachidonic acid in the granulosa cells of the cumulus. Thus, the expression of COX-2 in the cumulus cells promotes the synthesis of PGs, mainly prostaglandin E (PGE), and ensures the expansion of the cumulus. 18 25 26 However, cumulus expansion occurs only when the ITI enters the follicle. The TSG-6 and the proteoglycans brevican and versican, induced by high concentrations of LH and HA stabilization, are rapidly expressed in the cumulus granulosa cells of preovulatory follicles. 16 In the context of deficiency of the TSG-6 enzyme, the extracellular matrix is not structured, compromising cumulus expansion. 27 The PTX-3 protein, with an affinity for TSG6, is also responsible for the stability of the cumulus matrix. The interaction between these enzymes appears to be crucial for the structuring and expansion of the cumulus matrix, enabling the dispersion of the cumulus cells away from the oocyte. 20 Collectively, these observations indicate that HA, ITI, and COX-2, induced by the TSG-6 gene, are critical for cumulus matrix formation, cumulus cell differentiation, and, ultimately, cumulus expansion.

#### Oocyte Maturation

**Fig. 6:**
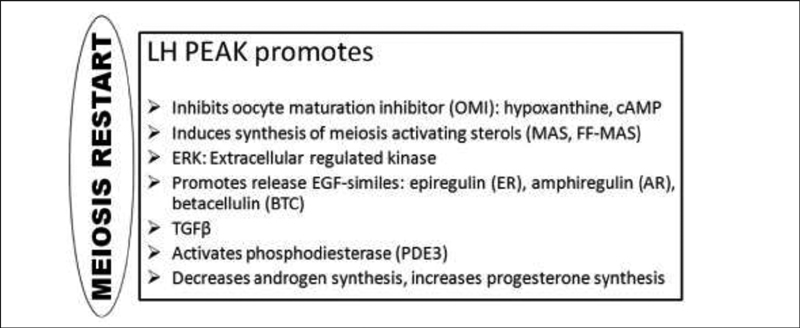
The role of the LH in meiosis resumption. **Source:** Coticchio et al. 28 Abbreviations: TGFβ, transforming growth factor β; EGF, epidermal growth factor.

**Fig. 7:**
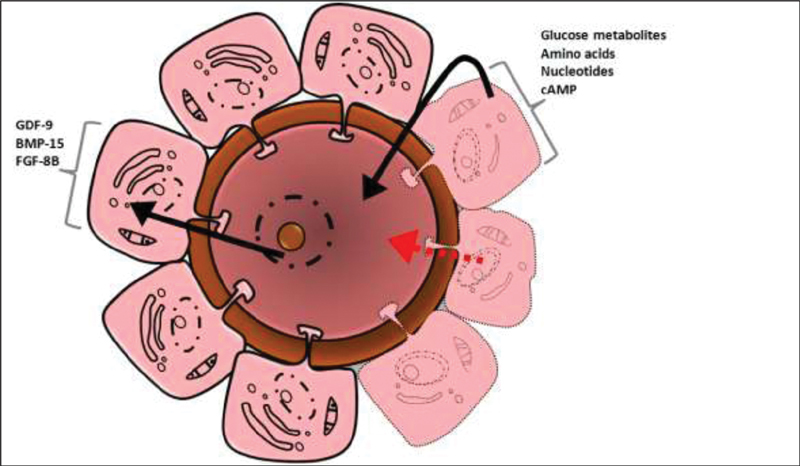
Cell-cell signaling between the oocyte and granulosa cells in the final stage of follicle development. **Source:** Adapted from: Sutton et al. 29 Abbreviations: GDF-9, growth differentiation factor 9; BMP-15, bone morphogenetic protein 15; FGF-8B, fibroblast growth factor 8B.

The oocyte maturation process aims to empower the female gamete and ensure its subsequent development until the activation of the embryonic genome occurs. Therefore, chromatin condensation is relevant in the continuity of meiosis, redistribution of organelles in the cytoplasm, and alterations in the cytoskeleton; all of these modifications are precisely regulated and coordinated ( Fig. 6 ). 28 For this to happen, there is paracrine cross-talk between the oocyte and cumulus cells. Cumulus cells penetrate the zona pellucida and limit the ooelema gap junction between the cumulus and the oocyte transfer of small molecules. 28 Biochemically, the oocyte regulates the metabolism of cumulus cells, which in turn provide ions, metabolites, amino acids, and small oocyte regulatory molecules ( Fig. 7 ). 29 Paracrine oocyte factors are soluble, and are generically referred to as oocyte-secreted factors (OSFs). 30 The growth differentiation factor 9 (GDF-9), the bone morphogenetic protein 15 (BMP15), and, to a lesser extent, the BMP6 are considered OSFs; all belong to the family of transforming growth factors β (TGFβ). 30 31 These factors coordinate the differentiation lineage and function of granulosa cells.

The functions of the OSFs include growth stimulation, prevention of apoptosis, inhibition of luteinization, regulation of energy metabolism, cholesterol biosynthesis, and regulation of cumulus expansion. 32 33 34 The factors that regulate the relationship between cumulus granulosa cells and the oocyte include ions, metabolites, amino acids, and small intracellular signaling molecules such as cAMP, cyclic guanosine monophosphate (cGMP), and inositol triphosphate-3 (IP3). 6 32 In the regulation of meiosis, cAMP synthesized by the oocyte itself and by cells of the mural granulosa and cumulus reaches the oocyte through the junctions of the hexameric lacunar canal composed of connectin proteins. 35 36

In general, the properties of lacunar junctions enable the direct and bidirectional transport of small molecules between the oocyte and the granulosa cells. High intraoocyte levels of cAMP maintain the oocyte in the stage of germ vesicle, through suppression of the activity of the maturation-proimoting factor (MPF). 37 38 39 Follicle somatic cells also provide cGMP to the oocyte, inhibiting the phosphodiesterase enzyme type 3A (PDE3A), thereby preventing the degradation of cAMP with the accumulation of this factor and inhibition of the resumption of meiosis. 38 40 With the LH stimulus at high concentrations, the connectins close, decreasing the contribution of cAMP and cGMP from the cumulus cells to the oocyte. Therefore, the decrease in cAMP levels leads to the phosphorylation of PDE3A that degrades the cAMP. The degradation of cAMP enables the synthesis of the MPF, which promotes the resumption of meiosis I. 41

In a recent study 42 in mice, the expression of natriuretic peptide type C (NPPC) was found in the mural granulosa cells, and natriuretic peptide receptor 2 (NPR2) was found in cumulus cells. With the communication between these two cell types the NPPC ligand and NPR2 stimulate the secretion of cGMP and cAMP. By adding NPPC to the culture media, an increase in the rates of oocytes that did not resume meiosis was observed, favoring the synchrony between nuclear maturation and cytoplasmic maturation. 42 During cytoplasmic maturation, there is a physical rearrangement of mitochondrial groups and endoplasmic reticulum, following the maturation time and energy dependence of the meiotic spindles so that chromatin is divided.

The meiotic spindles are responsible for the continuity of the meiotic division and extrusion of the two polar corpuscles. Initially, the mitochondrial groups are in a central position in the oocyte. As the maturation progresses, they migrate to the edges of the oocyte, close to the extruding regions of the polar body. 43 44 The MPF is the factor directly involved in cytoplasmic maturation, because, in addition to inducing the breakdown of the germ vesicle, it promotes the condensation of chromosomes, moving them from prophase I to metaphase I (MI), in which there is the formation of the meiotic spindle and the alignment of chromosomes in the center of the spindle. Then, anaphase I occurs, which consists of the separation of homologous chromosomes. Sequentially, telophase I begins with the extrusion of the first polar body, and the oocyte is in the metastasis II stage. At this stage, there is the formation of the second meiotic spindle and alignment of chromosomes, following anaphase II and telophase II and, finally, the extrusion of the second polar body. 45 46 The oocyte remains in this stage until ovulation occurs and there is the penetration of the sperm.

### Follicular Wall Digestion

**Fig. 8:**
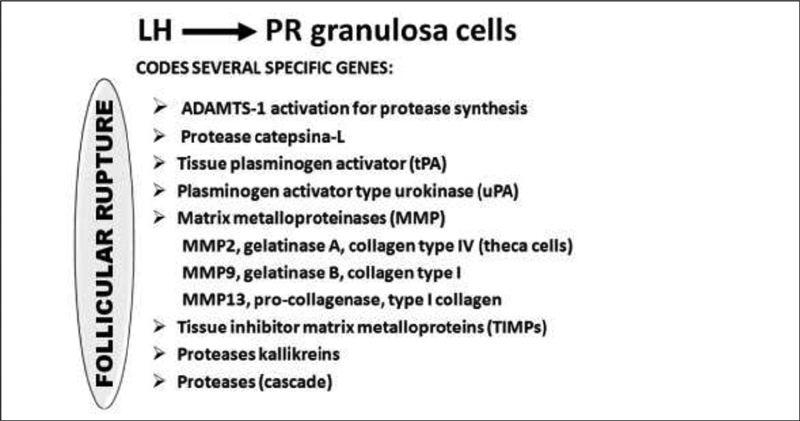
Role of LH-induced genes in the digestion of the follicular wall.

Morphological and biochemical changes that result in rupture of the follicular wall and oocyte extrusion occur basically by the action of the LH, because it induces the synthesis and secretion of various enzymes ( Fig. 8 ). The role of the FSH is smaller in this process, when the oocyte and cumulus cells are still fixed in the extracellular matrix (ECM). With the LH peak, LHR on the surface of the granulosa cells activates the digestion of the ECM within the theca layers and tunica albuginea at the ovarian surface via adenyl cyclase. The effectiveness of ECM digestion occurs through the balance between matrix components and proteases in the cumulus, oocyte, and endothelium cells that form the corpus luteum. 21 Theca cells express a variety of matrix metalloproteinases (MMPs), including MMP2 (gelatinase A), MMP9 (gelatinase B), MMP13 (collagenase), MMP14, MMP16, MMP19, and tissue inhibitor of MMPs-1 (TIMP-1). 9

The ADAMTS 16, present in luteinized granulosa cells, responds to FSH stimulation and actively participates in the process of structural follicle remodeling at the time of ovulation. The role of the LH on PR is mimicked by cAMP-inducing agonists (FSH, forskolin). Targets of PR appear to control the rupture of the follicle, mainly ADAMTS-1 (a disintegrin and metalloproteinase with thrombospondin) and cathepsin L. Among the proteases involved, thrombospondins 1 and 4 (ADAMTS1/4) promote the breakdown of the proteoglycan family structures, such as versican, through granulosa activation by PRs, 47 thereby contributing to the follicular rupture. Through its receptor in granulosa cells, the LH induces the transcription of early growth regulatory factor-1 (EGR-1), *CAAT* enhancer-binding protein β (C/EBPβ), PR, and other activator protein-1 family members (proto-oncogenes, c-Fos, c-Jun, Fra2, JunD), all involved in the functional activity of the granulosa cells of the ovulating follicle.

**Fig. 9:**
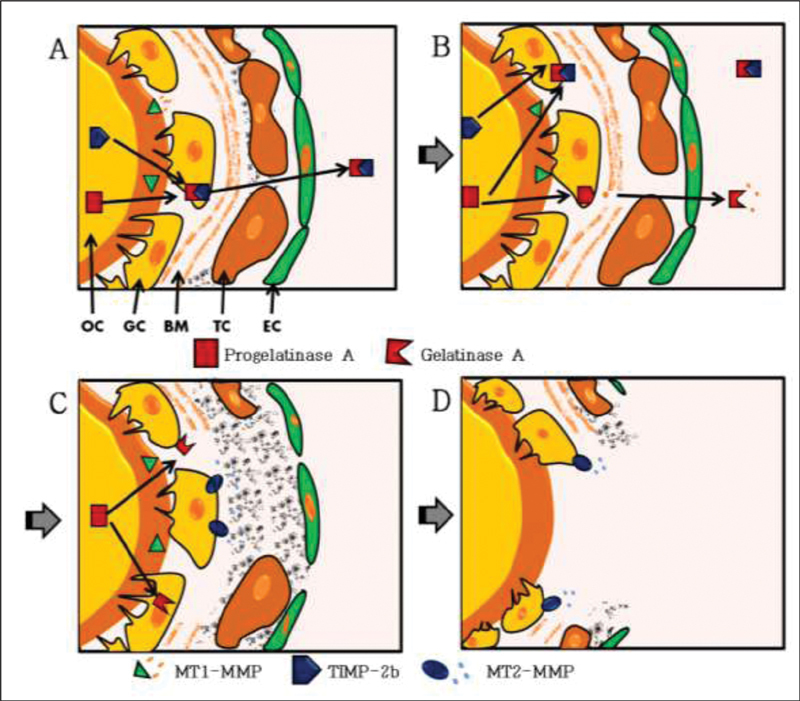
A model of follicle rupture during ovulation in the Japanese rice fish, also known as medaka. ( **A** ) In the follicle, a few hours before ovulation, progelatinase A is activated by membrane type 1-matrix metalloproteinase (MT1-MMP) on the surface of the oocyte, whereas gelatinase A is immediately inactivated by the tissue inhibitor of matrix metalloproteinases-2b (TIMP-2b). ( **B** ) At the time of ovulation, the hydrolysis of basement membrane type-IV collagen is initiated by active gelatinase A at the follicle–ovarian surface contact site. ( **C** ) membrane type 2-matrix metalloproteinase (MT2-MMP), which is now expressed on the surface of the granulosa cells, can degrade the type-I collagen that is present in the theca cell layer. ( **D** ) As a result, the oocyte is exposed at the contact site, leading to ovulation. Abbreviations: BM, basement membrane; EC, epithelial cell; GC, granulosa cell; OC, oocyte; TC, theca cell. Source: Ogiwara et al. 49 We would like to thank the National Academy of Sciences of the United States by permission

The proteoglycan (versican, brevican) components of the ECM induced by the LH peak, on either granulosa or theca cells, serve as substrates preferably for ADAMTS 1, culminating in follicular rupture. 47 Metalloproteinases such as plasminogen and collagenase are part of the follicular digestion process, and their control is mediated by metalloproteinase inhibitors, ensuring local homeostasis and completion of the ovulation process. 48 To illustrate, the model proposed by Ogiwara et al. 49 in the Japanese rice fish, also known as medaka, shows the involvement of proteinases in the lysis of the follicular wall ( Fig. 9 ).

After the rupture of the follicular wall, there is tissue reorganization by the activation of promatrix factors, which, in an organized and vascularized way, causes granulosa cell differentiation into luteal cells, thereby originating the corpus luteum. The corpus luteum is composed of functional cells for the synthesis of progesterone, the main regulator of the pituitary secretion of gonadotropins, the principal factors involved in the maintenance of the corpus luteum until initial gestation. 50 In the absence of maternal recognition of pregnancy, the corpus luteum regresses rapidly, and the ovarian cycle is resumed. 51

## References

[1] Russell D L Robker R L Molecular mechanisms of ovulation: co-ordination through the cumulus complex Hum Reprod Update 2007 13 03 289 312 10.1093/humupd/dml062 17242016

[2] Fraser I S Critchley H O Munro M G Broder M Can we achieve international agreement on terminologies and definitions used to describe abnormalities of menstrual bleeding? Hum Reprod 2007 22 03 635 643 10.1093/humrep/del478 17204526

[3] Latham K E Bautista F D Hirao Y O'Brien M J Eppig J J Comparison of protein synthesis patterns in mouse cumulus cells and mural granulosa cells: effects of follicle-stimulating hormone and insulin on granulosa cell differentiation in vitro Biol Reprod 1999 61 02 482 492 10.1095/biolreprod61.2.482 10411531

[4] Peng X R Hsueh A J LaPolt P S Bjersing L Ny T Localization of luteinizing hormone receptor messenger ribonucleic acid expression in ovarian cell types during follicle development and ovulation Endocrinology 1991 129 06 3200 3207 10.1210/endo-129-6-3200 1954899

[5] Haghighat N Van Winkle L J Developmental change in follicular cell-enhanced amino acid uptake into mouse oocytes that depends on intact gap junctions and transport system Gly J Exp Zool 1990 253 01 71 82 10.1002/jez.1402530110 2313243

[6] Albertini D F Combelles C M Benecchi E Carabatsos M J Cellular basis for paracrine regulation of ovarian follicle development Reproduction 2001 121 05 647 653 10.1530/rep.0.1210647 11427152

[7] de Medeiros S F Gil-Junior A B Barbosa J S Isaías E D Yamamoto M M New insights into steroidogenesis in normo- and hyperandrogenic polycystic ovary syndrome patients Arq Bras Endocrinol Metabol 2013 57 06 437 444 10.1590/s0004-27302013000600005 24030183

[8] van den Hurk R Zhao J Formation of mammalian oocytes and their growth, differentiation and maturation within ovarian follicles Theriogenology 2005 63 06 1717 1751 10.1016/j.theriogenology.2004.08.005 15763114

[9] Richards J S Russell D L Ochsner S Espey L L Ovulation: new dimensions and new regulators of the inflammatory-like response Annu Rev Physiol 2002 64 69 92 10.1146/annurev.physiol.64.081501.131029 11826264

[10] Schatten H Sun Q Y Centrosome dynamics during mammalian oocyte maturation with a focus on meiotic spindle formation Mol Reprod Dev 2011 78(10-11):757 768 10.1002/mrd.21380 21887720

[11] Espey L L Ovulation as an inflammatory reaction--a hypothesis Biol Reprod 1980 22 01 73 106 10.1095/biolreprod22.1.73 6991013

[12] Park J Y Su Y Q Ariga M Law E Jin S L Conti M EGF-like growth factors as mediators of LH action in the ovulatory follicle Science 2004 303(5658):682 684 10.1126/science.1092463 14726596

[13] Ashkenazi H Cao X Motola S Popliker M Conti M Tsafriri A Epidermal growth factor family members: endogenous mediators of the ovulatory response Endocrinology 2005 146 01 77 84 10.1210/en.2004-0588 15459120

[14] Shimada M Hernandez-Gonzalez I Gonzalez-Robayna I Richards J S Paracrine and autocrine regulation of epidermal growth factor-like factors in cumulus oocyte complexes and granulosa cells: key roles for prostaglandin synthase 2 and progesterone receptor Mol Endocrinol 2006 20 06 1352 1365 10.1210/me.2005-0504 16543407

[15] Sirard M A Desrosier S Assidi M In vivo and in vitro effects of FSH on oocyte maturation and developmental competence Theriogenology 2007 68 01 S71 S76 10.1016/j.theriogenology.2007.05.053 17588652

[16] Ochsner S A Day A J Rugg M S Breyer R M Gomer R H Richards J S Disrupted function of tumor necrosis factor-alpha-stimulated gene 6 blocks cumulus cell-oocyte complex expansion Endocrinology 2003 144 10 4376 4384 10.1210/en.2003-0487 12959984

[17] Joyce I M Pendola F L O'Brien M Eppig J J Regulation of prostaglandin-endoperoxide synthase 2 messenger ribonucleic acid expression in mouse granulosa cells during ovulation Endocrinology 2001 142 07 3187 3197 10.1210/endo.142.7.8268 11416041

[18] Hizaki H Segi E Sugimoto Y Hirose M Saji T Ushikubi F Abortive expansion of the cumulus and impaired fertility in mice lacking the prostaglandin E receptor subtype EP(2) Proc Natl Acad Sci U S A 1999 96 18 10501 10506 10.1073/pnas.96.18.10501 10468638 PMC17918

[19] Yamashita Y Hishinuma M Shimada M Activation of PKA, p38 MAPK and ERK1/2 by gonadotropins in cumulus cells is critical for induction of EGF-like factor and TACE/ADAM17 gene expression during in vitro maturation of porcine COCs J Ovarian Res 2009 2 20 10.1186/1757-2215-2-20 20034375 PMC2803446

[20] Richards J S Hernandez-Gonzalez I Gonzalez-Robayna I Teuling E Lo Y Boerboom D Regulated expression of ADAMTS family members in follicles and cumulus oocyte complexes: evidence for specific and redundant patterns during ovulation Biol Reprod 2005 72 05 1241 1255 10.1095/biolreprod.104.038083 15659705

[21] Gao S De Geyter C Kossowska K Zhang H FSH stimulates the expression of the ADAMTS-16 protease in mature human ovarian follicles Mol Hum Reprod 2007 13 07 465 471 10.1093/molehr/gam037 17519324

[22] Dozortsev D I Diamond M P Luteinizing hormone-independent rise of progesterone as the physiological trigger of the ovulatory gonadotropins surge in the human Fertil Steril 2020 114 02 191 199 10.1016/j.fertnstert.2020.06.016 32741458

[23] Richards J S Russell D L Robker R L Dajee M Alliston T N Molecular mechanisms of ovulation and luteinization Mol Cell Endocrinol 1998 145(1-2):47 54 10.1016/s0303-7207(98)00168-3 9922098

[24] Robker R L Russell D L Espey L L Lydon J P O'Malley B W Richards J S Progesterone-regulated genes in the ovulation process: ADAMTS-1 and cathepsin L proteases Proc Natl Acad Sci U S A 2000 97 09 4689 4694 10.1073/pnas.080073497 10781075 PMC18294

[25] Eppig J J Regulation by sulfated glycosaminoglycans of the expansion of cumuli oophori isolated from mice Biol Reprod 1981 25 03 599 608 10.1095/biolreprod25.3.599 6272891

[26] Calder M D Caveney A N Westhusin M E Watson A J Cyclooxygenase-2 and prostaglandin E(2)(PGE(2)) receptor messenger RNAs are affected by bovine oocyte maturation time and cumulus-oocyte complex quality, and PGE(2) induces moderate expansion of the bovine cumulus in vitro Biol Reprod 2001 65 01 135 140 10.1095/biolreprod65.1.135 11420233

[27] Fülöp C Szántó S Mukhopadhyay D Bárdos T Kamath V R Rugg M S Impaired cumulus mucification and female sterility in tumor necrosis factor-induced protein-6 deficient mice Development 2003 130 10 2253 2261 10.1242/dev.00422 12668637

[28] Coticchio G Dal-Canto M Guglielmo M C Mignini-Renzini M Fadini R Human oocyte maturation in vitro Int J Dev Biol 2012 56(10-12):909 918 10.1387/ijdb.120135gv 23417413

[29] Sutton M L Gilchrist R B Thompson J G Effects of in-vivo and in-vitro environments on the metabolism of the cumulus-oocyte complex and its influence on oocyte developmental capacity Hum Reprod Update 2003 9 01 35 48 10.1093/humupd/dmg009 12638780

[30] Gilchrist R B Ritter L J Armstrong D T Oocyte-somatic cell interactions during follicle development in mammals Anim Reprod Sci 2004 82-83 431 446 10.1016/j.anireprosci.2004.05.017 15271471

[31] Gilchrist R B Lane M Thompson J G Oocyte-secreted factors: regulators of cumulus cell function and oocyte quality Hum Reprod Update 2008 14 02 159 177 10.1093/humupd/dmm040 18175787

[32] Buccione R Schroeder A C Eppig J J Interactions between somatic cells and germ cells throughout mammalian oogenesis Biol Reprod 1990 43 04 543 547 10.1095/biolreprod43.4.543 2289008

[33] Eppig J J Wigglesworth K Pendola F Hirao Y Murine oocytes suppress expression of luteinizing hormone receptor messenger ribonucleic acid by granulosa cells Biol Reprod 1997 56 04 976 984 10.1095/biolreprod56.4.976 9096881

[34] Hussein T S Thompson J G Gilchrist R B Oocyte-secreted factors enhance oocyte developmental competence Dev Biol 2006 296 02 514 521 10.1016/j.ydbio.2006.06.026 16854407

[35] Teilmann S C Differential expression and localisation of connexin-37 and connexin-43 in follicles of different stages in the 4-week-old mouse ovary Mol Cell Endocrinol 2005 234(1-2):27 35 10.1016/j.mce.2004.10.014 15836950

[36] Gittens J E Kidder G M Differential contributions of connexin37 and connexin43 to oogenesis revealed in chimeric reaggregated mouse ovaries J Cell Sci 2005 118(Pt 21):5071 5078 10.1242/jcs.02624 16254245

[37] Aktas H Wheeler M B First N L Leibfried-Rutledge M L Maintenance of meiotic arrest by increasing [cAMP]i may have physiological relevance in bovine oocytes J Reprod Fertil 1995 105 02 237 245 10.1530/jrf.0.1050237 8568766

[38] Tsafriri A Chun S Y Zhang R Hsueh A J Conti M Oocyte maturation involves compartmentalization and opposing changes of cAMP levels in follicular somatic and germ cells: studies using selective phosphodiesterase inhibitors Dev Biol 1996 178 02 393 402 10.1006/dbio.1996.0226 8812137

[39] Bilodeau-Goeseels S Cows are not mice: the role of cyclic AMP, phosphodiesterases, and adenosine monophosphate-activated protein kinase in the maintenance of meiotic arrest in bovine oocytes Mol Reprod Dev 2011 78(10-11):734 743 10.1002/mrd.21337 21688336

[40] Tsafriri A Cao X Ashkenazi H Motola S Popliker M Pomerantz S H Resumption of oocyte meiosis in mammals: on models, meiosis activating sterols, steroids and EGF-like factors Mol Cell Endocrinol 2005 234(1-2):37 45 10.1016/j.mce.2004.09.009 15836951

[41] Gilchrist R B Smitz J EJ Thompson J G Current status and future trends of the clinical practice of human oocyte in vitro maturation Cambridge Cambridge University Press 2011 186 98

[42] Zhang M Su Y Q Sugiura K Xia G Eppig J J Granulosa cell ligand NPPC and its receptor NPR2 maintain meiotic arrest in mouse oocytes Science 2010 330(6002):366 369 10.1126/science.1193573 20947764 PMC3056542

[43] Dalton C M Carroll J Biased inheritance of mitochondria during asymmetric cell division in the mouse oocyte J Cell Sci 2013 126(Pt 13):2955 2964 10.1242/jcs.128744 23659999 PMC3699109

[44] Coticchio G Dal Canto M Renzini M M Guglielmo M C Brambillasca F Turchi D Oocyte maturation: gamete-somatic cells interactions, meiotic resumption, cytoskeletal dynamics and cytoplasmic reorganization Hum Reprod Update 2015 21 04 427 454 10.1093/humupd/dmv011 25744083

[45] Kishimoto H Hamada K Saunders M Backman S Sasaki T Nakano T Physiological functions of Pten in mouse tissues Cell Struct Funct 2003 28 01 11 21 10.1247/csf.28.11 12655146

[46] Sen A Caiazza F Oocyte maturation: a story of arrest and release Front Biosci (Schol Ed) 2013 5 451 477 10.2741/s383 23277062

[47] Russell D L Doyle K M Ochsner S A Sandy J D Richards J S Processing and localization of ADAMTS-1 and proteolytic cleavage of versican during cumulus matrix expansion and ovulation J Biol Chem 2003 278 43 42330 42339 10.1074/jbc.M300519200 12907688

[48] Curry T E Jr Dean D D Sanders S L Pedigo N G Jones P B The role of ovarian proteases and their inhibitors in ovulation Steroids 1989 54 05 501 521 10.1016/0039-128x(89)90044-5 2559499

[49] Ogiwara K Takano N Shinohara M Murakami M Takahashi T Gelatinase A and membrane-type matrix metalloproteinases 1 and 2 are responsible for follicle rupture during ovulation in the medaka Proc Natl Acad Sci U S A 2005 102 24 8442 8447 10.1073/pnas.0502423102 15941829 PMC1150835

[50] Kurita T Wang Y Z Donjacour A A Zhao C Lydon J P O'Malley B W Paracrine regulation of apoptosis by steroid hormones in the male and female reproductive system Cell Death Differ 2001 8 02 192 200 10.1038/sj.cdd.4400797 11313721

[51] Smith R K Carroll P M Allard J D Simon M A MASK, a large ankyrin repeat and KH domain-containing protein involved in Drosophila receptor tyrosine kinase signaling Development 2002 129 01 71 82 11782402 10.1242/dev.129.1.71

